# Continued Homelessness and Depressive Symptoms in Older Adults

**DOI:** 10.1001/jamanetworkopen.2024.27956

**Published:** 2024-08-15

**Authors:** Sarah K. Dobbins, Cheyenne M. Garcia, Jennifer L. Evans, Karen Valle, David Guzman, Margot B. Kushel

**Affiliations:** 1 School of Nursing, University of California, San Francisco; 2 Center for Vulnerable Populations, University of California, San Francisco; 3 UCSF Benioff Homelessness and Housing Initiative, University of California, San Francisco

## Abstract

**Importance:**

Depression is common in adults experiencing homelessness. It is unclear whether continued homelessness is associated with more depressive symptoms.

**Objective:**

To examine the association between residential status and depressive symptoms in adults aged 50 years or older experiencing homelessness at study entry.

**Design, Setting, and Participants:**

This cohort study analyzed results from the Health Outcomes of People Experiencing Homelessness in Older Middle Age (HOPE HOME) project, which in 2013 began enrolling adults aged 50 years or older experiencing homelessness in Oakland, California, and conducted structured interviews every 6 months for a mean duration of 5.5 years through 2023 (for this cohort study). Eligible participants included those aged 50 years or older, able to speak English, and experiencing homelessness at enrollment. We analyzed data collected from 2013 to 2023.

**Exposures:**

The exposure of interest was residential status. At follow-up visits, residential status was categorized as (1) homelessness (meeting the HEARTH [Homeless Emergency Assistance and Rapid Transition to Housing] Act definition) or (2) housed (living in a noninstitutional environment and not meeting the HEARTH Act definition).

**Main Outcomes and Measures:**

The primary outcome was moderate to severe depressive symptoms (with Center for Epidemiologic Studies–Depression [CES-D] scale score ≥22). The augmented inverse probability of treatment weighting (AIPTW) approach was used to examine the association between continued homelessness and depressive symptoms. The AIPTW adjusted for the following variables: number of chronic health conditions, age, sex, visiting a health care practitioner, receiving outpatient mental health treatment, receiving mental health medication, exposure to abuse, substance use disorder, and binge drinking.

**Results:**

The cohort was composed of 450 participants, of whom 343 (76.2%) were males, and the mean (SD) age was 58.5 (5.2) years. Participants completed a median (IQR) of 8.9 (8-11) follow-up visits. With 1640 person-years of observation time, participants continued homelessness for 880 person-years (57.1%) and experienced being housed for 715 person-years (44.3%). Many participants (304 [78.0%]) were housed during at least 1 follow-up visit. The odds of a CES-D scale score of 22 or higher was significantly higher among participants who continued experiencing homelessness than among housed participants (marginal causal odds ratio, 1.08; 95% CI, 1.04-1.11; *P* < .001).

**Conclusions and Relevance:**

This cohort study found that continued homelessness was associated independently with increased odds of depressive symptoms. Obtaining housing may have a favorable role in depression and overall well-being of older adults experiencing homelessness and may be considered as a mental health intervention.

## Key Points

**Question:**

Is continued homelessness (vs regaining housing) associated with increased depressive symptoms among older adults?

**Findings:**

In a cohort study of 450 adults aged 50 years or older who were experiencing homelessness at baseline, the odds of experiencing moderate to severe depressive symptoms were significantly higher among participants with continued homelessness compared with those who regained housing.

**Meaning:**

The findings of this study suggest that housing has a favorable role in depression and overall well-being of older adults experiencing homelessness and may be considered as a mental health intervention.

## Teaser

This cohort study evaluates the association between residential status and moderate to severe depressive symptoms over a 5-year period in individuals aged 50 years or older experiencing homelessness.

## Introduction

People experiencing homelessness have a higher prevalence of depressive symptoms than the general population (38%-56% vs 5%-7%), but the directionality is unclear.^1,2^ Depressive symptoms may be associated with homelessness via loss of work and income and adverse implications for social relationships. Homelessness could play a role in worsened depressive symptoms through environmental exposures (eg, abuse, sleep disturbances) or interference with access to mental health treatment.^3^ While most studies of homelessness are cross-sectional, homelessness is dynamic, with frequent entrances and exits.^4^

Half of the single adults experiencing homelessness in the US are aged 50 years or older; this proportion is expected to increase.^5,6^ This homeless population experiences a higher prevalence of geriatric conditions such as falls, urinary incontinence, depression, and functional and cognitive impairments compared with adults 20 to 30 years older in the general population.^7^ Homelessness may be associated with accelerated aging through interactions with biological, social, and environmental factors, including depression.^7,8,9,10^ Therefore, adults experiencing homelessness are functionally an older adult population at age 50 years.^7^ Depression among older adults experiencing homelessness may exacerbate co-occurring chronic diseases, substance use disorders (SUDs), and geriatric syndromes, such as functional and cognitive impairment.^10^

Therefore, we examined the association between residential status and depressive symptoms in a longitudinal cohort of adults aged 50 years or older who were experiencing homelessness at study entry. We hypothesized that those who continued experiencing homelessness would have higher odds of moderate to severe depressive symptoms compared with those who regained housing.

## Methods

### Participants and Procedures

The Health Outcomes of People Experiencing Homelessness in Older Middle Age (HOPE HOME) is an ongoing longitudinal cohort study of the health and life course events among older adults experiencing homelessness. Participants are interviewed at baseline and at 6-month intervals at a community-based site. The University of California, San Francisco Institutional Review Board approved this study. Verbal informed consent was obtained from all participants. We followed the STROBE reporting guideline.

Between July 2013 and June 2014, we used purposive venue-based sampling to recruit 350 participants aged 50 years or older experiencing homelessness in Oakland, California (wave 1).^11,12^ Between August 2017 and July 2018, using the same methods, we recruited an additional 100 participants aged 53 years or older (wave 2). Eligibility criteria included (1) homelessness, as defined by the HEARTH (Homeless Emergency Assistance and Rapid Transition to Housing) Act; (2) ability to speak English; (3) meeting the age criteria; and (4) ability to provide informed consent using a teach-back method.^13,14^ The HEARTH Act defines someone as experiencing homelessness if they lack a fixed residence, reside in a place not typically used for sleeping, are fleeing domestic violence, or are at imminent risk of losing housing within 14 days.^13^

We selected a sample from 5 low-cost meal programs, 5 overnight shelters, 1 recycling center, and places where unsheltered people stayed. For this analysis, we included the first 66 months of study visits for each participant (baseline through month 60) through 2023. We began the analysis in May 2021 and finished post hoc analysis in May 2024.

### Outcome

We measured depressive symptoms using the Center for Epidemiologic Studies–Depression (CES-D) scale at baseline and follow-up visits. The CES-D scale is a 20-item self-assessment of past-week depressive symptoms; the scale, validated in people experiencing homelessness, takes 2 to 5 minutes to complete.^15,16,17,18^ Responses to the items vary from rarely or none of the time (<1 day) to most or all of the time (5-7 days), and scores range from 0 to 3, with higher scores indicating higher severity. We defined depression on the CES-D scale as a binary variable using a cutoff score of 22 or higher to indicate moderate to severe depressive symptoms.

### Exposure

The exposure of interest was residential status. All participants were experiencing homelessness at study entry. At follow-up visits, we categorized residential status as homelessness (meeting the HEARTH Act definition) or housed or regained housing (living in a noninstitutional environment and not meeting the HEARTH Act definition).^13^

### Other Variables

At baseline, we measured age, sex, race and ethnicity, age at first experience of homelessness, chronic conditions, SUD, exposure to abuse, health care engagement, and mental health treatment (Table 1). We categorized race and ethnicity as Black or African American (hereafter, Black); Latina, Latino, or Latinx; White; and multiracial or other (including Asian American or Pacific Islander, Middle Eastern, Native American or American Indian, or multiracial). We assessed race as a proxy for exposure to anti-Black racism, which could affect both housing status and depressive symptoms.^19,20,21^ During each interview, we asked participants whether a health care practitioner ever diagnosed them with myocardial infarction, congestive heart failure, stroke, diabetes, chronic obstructive pulmonary disease or asthma, HIV/AIDS, liver disease, or cancer.^22^

**Table 1.** Baseline Characteristics of Participants

Characteristic	Participants, No. (%)		
	Total (n = 450)	CES-D scale score <22 (n = 278)	CES-D scale score ≥22 (n = 164)
Age, y	58.5 (5.2)	59.4 (5.4)	57.0 (4.4)
50-59	267 (59.2)	149 (55.8)	118 (44.2)
≥60	175 (38.8)	129 (73.7)	46 (26.3)
Sex			
Male	343 (76.2)	212 (76.3)	124 (75.6)
Female	107 (23.8)	66 (23.7)	40 (24.4)
Race and ethnicity^a^			
Black or African American	360 (80.0)	231 (83.1)	123 (75.0)
Latina, Latino, Latinx	21 (4.7)	11 (4.0)	10 (6.1)
White	48 (10.7)	23 (8.3)	23 (14.0)
Multiracial or other^b^	21 (4.7)	13 (4.7)	8 (4.9)
Age at first experience of homelessness, y			
18-25	65 (14.6)	29 (10.6)	35 (21.5)
26-49	183 (41.0)	106 (38.6)	73 (44.8)
50-59	151 (33.9)	101 (36.7)	47 (28.8)
≥60	47 (10.5)	39 (14.2)	8 (4.9)
No. of chronic conditions	1.5 (1.4)	1.3 (1.3)	1.8 (1.5)
0-1	262 (58.2)	175 (63.0)	84 (51.2)
≥2	188 (41.8)	103 (37.1)	80 (48.8)
Binge drinking			
Yes	47 (10.5)	21 (7.6)	26 (15.9)
No	400 (89.5)	254 (92.4	138 (84.1)
Moderate- to high-risk SUD			
Yes	181 (40.2)	100 (36.0)	80 (48.8)
No	269 (59.8)	178 (64.0)	84 (51.2)
Exposure to abuse			
Any	96 (21.4)	41 (14.8)	51 (31.1)
None	353 (78.6)	237 (85.2)	113 (68.9)
Physical	49 (11.0)	18 (6.5)	30 (18.3)
Verbal	71 (15.9)	30 (10.8)	38 (23.2)
Sexual	8 (1.8)	3 (1.1)	5 (3.1)
Visited a health care practitioner			
Yes	258 (57.3)	160 (57.6)	95 (57.9)
No	192 (42.7)	118 (42.4)	69 (42.1)
Received outpatient mental health treatment			
Yes	72 (16.0)	29 (10.4)	42 (25.6)
No	378 (84.0)	249 (89.6)	122 (74.4)
Prescribed mental health medications			
Yes	97 (21.6)	36 (13.0)	59 (36.0)
No	353 (78.4)	242 (87.0)	105 (64.0)

Abbreviations: CES-D, Center for Epidemiologic Studies–Depression (score of ≥22 indicates moderate to severe depressive symptoms); SUD, substance use disorder.

^a^ Race and ethnicity were self-identified by participants at interviews.

^b^ Multiracial or other category included Asian American or Pacific Islander, Middle Eastern, Native American or American Indian, or multiracial.

To ascertain moderate- to high-risk drug use (cocaine, amphetamines, and opioids) in the past 6 months, we used the World Health Organization Alcohol, Smoking, and Substance Involvement Screening Test score of 4 or higher.^23^ We operationalized binge drinking as consuming 6 or more alcoholic drinks on 1 occasion at least monthly.

We asked participants whether they visited a health care practitioner at their usual (non–emergency department) place of care, received outpatient mental health treatment, and received a prescription for a medication for any psychological or emotional problems in the past 6 months. We measured physical, verbal, and sexual abuse in prior 6 months.^24^ We assessed social support by the number of confidants (0, 1-5, or ≥6).^25,26^

### Statistical Analysis

To estimate the association between residential status and depression, we used the causal framework of augmented inverse probability of treatment weighting (AIPTW). The AIPTW is a statistical approach that combines regression and inverse probability weighing using 2 models for the outcome of depressive symptoms: 1 for the unexposed condition, and 1 for the exposed condition. The contrast of these 2 models provides the mean treatment effect. The AIPTW approach estimates a marginal causal odds ratio (MCOR), which is the mean outcome of moving an entire population from the unexposed group (housed) to the exposed group (homelessness) while adjusting for covariates at each time point.

The advantages of AIPTW include reduced bias in the association and being doubly robust, meaning that it is consistent as long as either the outcome model is correct or the propensity score model is correctly specified and is therefore less sensitive to unmeasured confounding.^27,28^ Another advantage is that AIPTW accounts for time variability of both exposure and outcome.^29,30^ We considered residential status to be a time-varying exposure, as participants exited out of and re-entered homelessness. Residential status is a nonrandom event; some individuals experiencing homelessness may be more likely to regain housing based on observed and unobserved variables. Using AIPTW, we analyzed changes in depressive symptoms as participants moved in and out of housing, while controlling for the confounding covariates related to both outcome and exposure.^29,30^ The AIPTW approach estimates a cross-sectional association at each time point to account for time-varying exposure, outcome, and covariates. Mean treatment effects are estimated by averaging the association across the study. Therefore, the MCOR is a time-varying cross-sectional association.

We first examined the unadjusted, unweighted associations among the exposure, outcome, and other variables using random-effects logistic regression with SEs clustered on individuals (Table 2). Using clustered SEs treats each participant’s repeated measures as a cluster and accounts for the correlation of repeated measures in the same individual.^31^ We used AIPTWs to estimate the MCOR for residential status with moderate to severe depressive symptoms. The AIPTWs were conditioned on time-invariant variables (total number of chronic health conditions, age at first experience of homelessness, age at baseline, sex) and time-varying variables (visited a health care practitioner, received outpatient mental health treatment, prescribed mental health medications, exposure to abuse, SUD, binge drinking). The same set of covariates was used for both the exposure and outcome models. We selected these covariates a priori based on a review of current literature. We did not use time-lagged variables because measures at each visit reflect the past 6 months, providing sufficient time to produce a change in depressive symptoms.^32^ We set α = .05 (*P* < .05) to indicate statistical significance for all analyses and performed all analyses in Stata, version 15 (StataCorp LLC).^33^ We excluded 35 observations (1.2%) wherein participants were living in skilled nursing facilities (SNFs). For more information, see the eMethods in Supplement 1.

**Table 2.** Factors Associated With Moderate to Severe Depressive Symptoms During the Study Period (n = 450 Participants)

Variable	CES-D scale score ≥22	
	OR (SE)	*P* value
Residential status		
Housed, noninstitutional	1 [Reference]	NA
Homelessness	1.44 (0.16)	.002
Skilled nursing facility	1.09 (0.49)	.85
Age, y	0.95 (0.02)	.004
Male vs female	1.52 (0.27)	.02
Race and ethnicity^a^		
Black or African American	1 [Reference]	NA
Latina, Latino, or Latinx	2.49 (0.91)	.01
White	1.38 (0.36)	.23
Multiracial or other^b^	1.45 (0.49)	.27
Age at first experience of homelessness, y		
18-25	1 [Reference]	NA
26-49	0.65 (0.14)	.05
50-59	0.53 (0.12)	.005
≥60	0.28 (0.10)	<.001
No. of chronic conditions		
0-1	1 [Reference]	NA
≥2	1.45 (0.23)	.02
Binge drinking	1.84 (0.32)	.001
Moderate- to high-risk SUD	1.83 (0.28)	<.001
Any exposure to abuse	2.95 (0.32)	<.001
Physical	2.79 (0.42)	<.001
Verbal	2.89 (0.32)	<.001
Sexual	4.44 (1.75)	<.001
Visited a health care practitioner	0.95 (0.11)	.67
Received outpatient mental health treatment	2.38 (0.35)	<.001
Prescribed mental health medications	3.58 (0.53)	<.001

Abbreviations: CES-D, Center for Epidemiologic Studies–Depression (score of ≥22 indicates moderate to severe depressive symptoms); NA, not applicable; OR, odds ratio; SUD, substance use disorder.

^a^ Race and ethnicity were self-identified by participants at interviews.

^b^ Multiracial or other included Asian American or Pacific Islander, Middle Eastern, Native American or American Indian, or multiracial.

#### Risk-of-Bias and Post Hoc Analyses

We performed analyses to examine risk of bias and the reliability of the results. First, we assessed whether we underestimated the probability of having depressive symptoms due to incomplete study follow-up or mortality. We examined depressive symptoms in 3 groups: (1) those with complete follow-up, (2) those who had died during follow-up, and (3) those who had not died but had missed at least 1 study visit. We used random-effects regression to examine whether those who had died or missed visits were more or less likely to experience increased depressive symptoms than those with complete follow-up. Second, we performed a restricted analysis using data from only wave-1 participants to account for possible bias from attrition and accumulation of deaths (n = 348) (eTable 1 in Supplement 1). Third, to examine the possibility of bias attributed to use of outpatient mental health services, we tested our hypotheses in a restricted subsample of participants who never accessed outpatient mental health services (n = 238) (eTable 2 in Supplement 1). Fourth, we included observations of participants living in SNFs to determine risk of bias from exclusion (eTable 3 in Supplement 1).

Fifth, we examined the implication of residential status for continuous CES-D scores (eTable 4 in Supplement 1). Sixth, we used a lagged variable for homelessness in the prior 6 months to examine temporality (eTable 5 in Supplement 1). Seventh, we used a lagged variable for depressive symptoms in the prior 3 study visits to examine the outcome of a depressive symptoms pattern preceding residential status (eTable 6 in Supplement 1). To address issues related to unmeasured confounding, we calculated an E value for the MCOR, which indicates the confounding strength capable of attenuating the association to one that is no longer significant.^34^

We conducted post hoc analyses to explore additional questions. We examined the association between residential status and depression with a time-varying social support variable (eTable 7 in Supplement 1).^25,26^ Social support may increase vulnerability to homelessness due to structural and economic factors, exposure to abuse, and mental health.^35^

## Results

### Sociodemographic and Health Characteristics

In this cohort study of 450 participants, there were 343 males (76.2%) and 107 females (23.8%) with a mean (SD) age of 58.5 (5.2) years (Figure; Table 1). Most participants identified as Black (360 [80.0%]), followed by 48 (10.7%) who identified as White; 21 (4.7%) as Latina, Latino, or Latinx; and 21 (4.7%) as multiracial or other. Nearly half of the cohort (198 [44.0%]) had their first experience of adult homelessness at the age of 50 years or older (Table 1). Forty-seven participants (10.5%) reported binge drinking in the past 6 months, and 181 (40.2%) met the criteria for moderate to high-risk SUD in the past 6 months.

**Figure.**
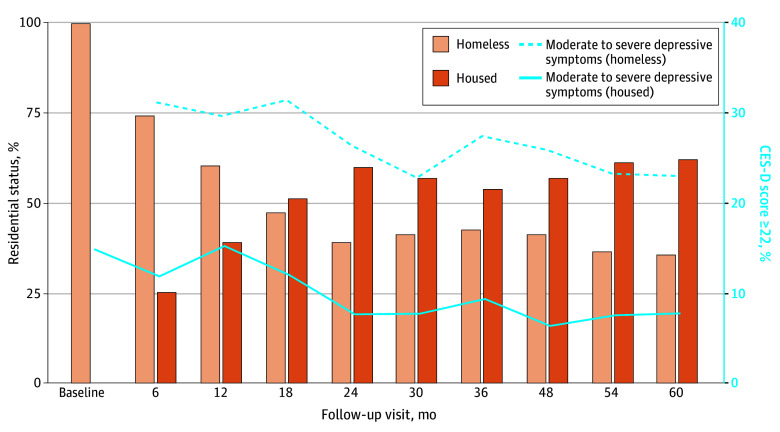
Proportion of Participants Experiencing Homelessness and Housed (Noninstitutional) Participants With Moderate to Severe Depressive Symptoms During Follow-Up CES-D indicates Center for Epidemiologic Studies–Depression scale.

At baseline, the mean (SD) number of chronic conditions was 1.5 (1.4). Nearly a third of participants (131 [29.1%]) reported liver disease; 163 (36.2%), chronic obstructive pulmonary disease or asthma; 115 (25.6%), diabetes; 74 (16.4%), stroke; 56 (12.4%), congestive heart failure; 48 (10.7%), cancer; 46 (10.2%), myocardial infarction; and 26 (5.8%), HIV/AIDS. Ninety-six individuals (21.0%) reported experiencing physical, verbal, or sexual abuse in the past 6 months.

In the prior 6 months, the majority of participants (258 [57.3%]) visited a health care practitioner at their usual place of care, some (72 [16.0%]) received outpatient mental health treatment from a mental health clinic, and one-fifth (97 [21.6%]) were prescribed mental health medications. The median (IQR) number of follow-up visits was 8.9 (8-11). Sixty participants (13.3%) completed only 1 study visit, and 161 (35.8%) lived and completed 10 to 11 study visits (eFigure 1 in Supplement 1). Ninety-two individuals (20.4%) died before they completed all follow-up visits (eFigure 2 in Supplement 1).

### Residential Status and Depressive Symptoms

This analysis included 1640 person-years of observation time; participants continued homelessness for 880 person-years (57.1%) and experienced being housed for 715 person-years (44.3%). During at least 1 follow-up visit, 332 participants (85.1%) experienced homelessness and 304 (78.0%) were housed. Homelessness exits included exits to permanent supportive housing, a rented apartment or house with or without rental subsidies, and a stable living arrangement with family or friends.

At baseline, the mean (SD) CES-D scale score was 18.4 (12.5), 226 individuals (51.1%) had clinically significant depressive symptoms, and 164 individuals (36.4%) had moderate to severe depressive symptoms. Cronbach α for the CES-D scale items ranged from 0.89 to 0.90 over the analysis period. Unadjusted associations between covariates and moderate to severe depressive symptoms are reported in Table 2. For example, moderate- to high-risk SUD, exposure to abuse, receiving outpatient mental health treatment, and being prescribed mental health medications were all associated with increased odds of moderate to severe depressive symptoms. We found that those experiencing homelessness compared with housed participants (referent) had significantly higher odds of moderate to severe depressive symptoms (MCOR, 1.08, 95% CI, 1.04-1.11; *P* < .001). We conducted a covariate balance summary (eTable 8 and eFigure 4 in Supplement 1).

### Risk-of-Bias Analyses

One hundred twenty-five participants (27.8%) lived and completed all 11 follow-up visits, 92 (20.4%) died during the analysis period, and half (233 [51.8%]) missed at least 1 study visit. We found no significant difference in depressive symptoms between these groups. The restricted analysis of the wave 1 cohort revealed higher odds of moderate to severe depressive symptoms (MCOR, 1.07; 95% CI, 1.02-1.11; *P* = .005) among those who were experiencing homelessness compared with those housed (eTable 1 in Supplement 1). The restricted analysis of those who never accessed outpatient mental health treatment revealed higher odds of moderate to severe depressive symptoms among those experiencing homelessness compared with those housed (MCOR, 1.06; 95% CI, 1.01-1.11; *P* = .03) (eTable 2 in Supplement 1). When we included observations wherein participants were staying in a SNF, the magnitude of the association changed negligibly (MCOR, 1.05; 95% CI, 0.97-1.14; *P* = .25) (eTable 3 in Supplement 1). When we used a lagged variable for homelessness status, the result was changed minimally (MCOR, 1.05; 95% CI, 1.01-1.90; *P* = .02) (eTable 5 in Supplement 1). The E value point estimate for our primary outcome was 1.37 (eFigure 3 in Supplement 1). We controlled for several major confounding variables, which decreased the likelihood that an unmeasured confounder would meet the threshold to fully explain the exposure-outcome association.^36^

### Post Hoc Analyses

We found that homelessness was associated with higher continuous CES-D scale scores (3.05; 95% CI, 2.00-4.13; *P* < .001) (eTable 4 in Supplement 1). At baseline, 143 individuals (31.8%) reported 0 confidants, 270 (60.0%) reported 1-5 confidants, and 37 (8.2%) reported 6 confidants. Those who were experiencing homelessness compared with those housed remained at higher odds of moderate to severe depressive symptoms while controlling for social support (MCOR, 1.06; 95% CI, 1.02-1.10; *P* < .01) (eTable 7 in Supplement 1).

## Discussion

This cohort study showed that continued homelessness (compared with regaining housing) was associated with higher odds of having moderate to severe depressive symptoms. Homelessness is dynamic, with frequent exits to housing and re-entrances to homelessness. Depressive symptoms vary over time with changes in life circumstances, mental health treatment, and environmental factors.^37^ Although the magnitude of this association was modest, it suggests a clinically relevant change in depressive symptoms. In marginalized populations, such as older adults experiencing homelessness, even a modest decrease in depressive symptoms can have downstream implications for physical health and quality of life.

Older adults experiencing homelessness have a high prevalence of depressive symptoms,^38,39^ which may be due to the high prevalence of other conditions in the homeless populations, such as chronic disease, exposure to abuse, and SUD.^1^ Limited agency among people experiencing homelessness, which facilitates an external locus of control, may contribute to depressive symptoms.^40,41^ Whether in sheltered or unsheltered settings, people experiencing homelessness must navigate stressful material deprivation, loss of sleep, exposure to violence, stigma, and shame, all of which may be factors in increased depressive symptoms.^42,43,44^ However, results of our analysis suggest that continued homelessness is associated with depressive symptoms, independent of other factors common in populations experiencing homelessness, supporting our hypotheses. The bias analyses suggest that this association is not significantly affected by bias.

The present work extends prior research on the prevalence of depressive symptoms in older adults by finding an independent association between experiencing homelessness and odds of experiencing moderate to severe depressive symptoms while accounting for housing as a time-varying exposure for over 5.5 years in a large sample of adults aged 50 years or older experiencing homelessness. Prior longitudinal studies had smaller sample sizes, selected participants based on their mental health condition or access to social or housing services (eg, homeless in a shelter, having a case manager, or enrollment in the US Department of Housing and Urban Development–Veterans Affairs Supportive Housing program), and had shorter follow-up.^7,32,37,45,46,47^ Many older adults experiencing homelessness do not have access to homelessness and housing services or health care services and thus do not receive a mental health diagnosis.^3^ Despite the growing population of older adults experiencing homelessness, much of the prior research on residential status and depressive symptoms involved younger adults experiencing homelessness.^1^ The poor health and functional status of older adults experiencing homelessness heighten the risk of depressive symptoms and their adverse implications for overall health, functional and cognitive impairment, and increased mortality.^48^

We followed up rehoused participants with many housing types, while other studies analyzed outcomes for 1 housing type.^45,46,47^ We followed up participants after they were housed, including those who subsequently lost housing. The within-person variation of residential status with depressive symptoms present in our study provides credence to these findings. To our knowledge, this study was the first to use AIPTWs to examine the association between residential status and depressive symptoms. The AIPTW analytical method accounted for the dynamic nature of homelessness through the time-varying, nonrandom exposure of residential status. We treated residential status as nonrandom because certain characteristics and experiences were associated with attaining housing. Some forms of social and structural support increased the odds of housing placement, such as having a case manager, receiving a housing subsidy, or functional impairment.^49^ We measured and accounted for possible confounding variables, which increased the reliability of the association. Because the AIPTW approach allowed individuals to remain in the analysis regardless of the number of follow-up visits, we examined this association with a larger sample size.

### Limitations

This study has several limitations. Because the study was observational, we could not prove causation. However, the AIPTW approach is a causal framework and provides support for a causal association between depression and homelessness.^50^ While it is possible that unmeasured confounding may change the results, we used a method that was doubly robust to confounding and included major confounders and time-varying covariates in the analysis. The bias and post hoc analyses increased the reliability of the conclusions. While complex aspects of social support may be a source of unmeasured confounding, the post hoc analysis suggests the association of depression and homelessness exists regardless of social support. We did not explore mediating variables in this study; therefore, we do not know the mechanisms for the association between housing and depressive symptoms.

## Conclusions

In this longitudinal cohort study of adults aged 50 years or older who were experiencing homelessness at study enrollment, continued homelessness vs regaining housing was associated with increased odds of moderate to severe depressive symptoms, independent of other factors. Housing may have a favorable role in depression and overall well-being of older adults experiencing homelessness and can be considered a therapeutic mental health intervention in aging.

## Notes

Supplement 1.
eMethods
**eTable 1.** Effect of Residential Status on Odds of Moderate-Severe Depressive Symptoms (CES-D ≥22) in HOPE-HOME 1 (n=348) **eTable 2.** Effect of Residential Status on Odds of Moderate-Severe Depressive Symptoms (CES-D ≥22) and Outpatient Mental Health Services in HOPE-HOME 1 & 2 **eTable 3.** Effect of Residential Status on Odds of Moderate-Severe Depressive Symptoms (CES-D ≥22) in HOPE-HOME 1 & 2 (n=444) **eTable 4.** Effect of Residential Status on Continuous CES-D Depression Score in HOPE-HOME 1 & 2 (N=444) **eTable 5.** Effect of Lagged Residential Status on Odds of Moderate-Severe Depressive Symptoms (CES-D ≥22) in HOPE-HOME 1 (n=348) **eTable 6.** Effect of Residential Status on Odds of Moderate-Severe Depressive Symptoms (CES-D ≥22) with Trends in Depression in HOPE-HOME 1 & 2 (n=444) **eTable 7.** Effect of Residential Status on Odds of Moderate-Severe Depressive Symptoms (CES-D ≥22) with Social Isolation Covariate in HOPE-HOME 1 & 2 (n=444) **eTable 8.** Covariate Balance Summary **eFigure 1.** Number of Follow-up Study Visits Completed **eFigure 2.** Analysis Sample Diagram **eFigure 3.** E-Value Curve **eFigure 4.** Density Plot of Propensity Scores in AIPTW Treatment Effects Model

Supplement 2.
Data Sharing Statement
