# Supplementary material for: Continued Homelessness and Depressive Symptoms in Older Adults
Source: JAMA Netw Open. 2024 Aug 15;7(8):e2427956. doi: 10.1001/jamanetworkopen.2024.27956 (PMC11327886; doi:10.1001/jamanetworkopen.2024.27956)

Supplemental Online Content

Dobbins SK, Garcia CM, Evans JL, Valle K, Guzman D, Kushel MB. Continued homelessness and depressive symptoms in older adults. *JAMA Netw Open*. 2024;7(8):e2427956. doi:10.1001/jamanetworkopen.2024.27956

eMethods

eTable 1. Effect of Residential Status on Odds of Moderate-Severe Depressive Symptoms (CES-D ≥ 22) in HOPE-HOME 1 (n=348)

eTable 2. Effect of Residential Status on Odds of Moderate-Severe Depressive Symptoms (CES-D≥22) and Outpatient Mental Health Services in HOPE-HOME 1 & 2

eTable 3. Effect of Residential Status on Odds of Moderate-Severe Depressive Symptoms (CES-D ≥ 22) in HOPE-HOME 1 & 2 (n=444)

eTable 4. Effect of Residential Status on Continuous CES-D Depression Score in HOPE-HOME 1 & 2 (N=444)

eTable 5. Effect of Lagged Residential Status on Odds of Moderate-Severe Depressive Symptoms (CES-D ≥ 22) in HOPE-HOME 1 (n=348)

eTable 6. Effect of Residential Status on Odds of Moderate-Severe Depressive Symptoms (CES-D ≥ 22) with Trends in Depression in HOPE-HOME 1 & 2 (n=444)

eTable 7. Effect of Residential Status on Odds of Moderate-Severe Depressive Symptoms (CES-D ≥ 22) with Social Isolation Covariate in HOPE-HOME 1 & 2 (n=444)

eTable 8. Covariate Balance Summary

eFigure 1. Number of Follow-up Study Visits Completed

eFigure 2. Analysis Sample Diagram

eFigure 3. E-Value Curve

eFigure 4. Density Plot of Propensity Scores in AIPTW Treatment Effects Model

© 2024 Dobbins SK et al. *JAMA Network Open*

This supplemental material has been provided by the authors to give readers additional information about their work.

© 2024 Dobbins SK et al. *JAMA Network Open*

eMethods

Below is a detailed description of the methods applied in our study of moderate-severe depressive symptoms and homelessness.

To estimate causal effects, the gold standard in research is the randomized control trial (RCT). However, it is unethical to randomize research participants to experience homelessness or being housed. Therefore, homelessness is often studied using observational studies. In longitudinal observational studies, participants are observed over time without intervention. The exposed and unexposed groups are not perfectly comparable in an observational study, as they would be in an RCT, due to possible confounding factors. Researchers can use statistical methods to adjust for this confounding and, therefore, can examine causal relationships between exposed and unexposed groups. An example of this, and the method we used in this study, is Inverse Probability of Treatment Weighting (IPTW).

In our study, the exposed and unexposed groups are based on residential status: homeless (exposed) and housed (unexposed). We used Augmented IPTW (AIPTW), which combines IPTW and average treatment effects (ATEs), to estimate the odds of moderate-severe depressive symptoms among older adults who remain homeless compared to those who regain housing over 5.5 years of follow-up. Using the AIPTW approach, differences in the outcome between groups can be attributed to an association with the exposure (homelessness) and the estimates may be interpreted as a causal association. We describe this in more detail below.

Inverse Probability of Treatment Weighting (IPTW)

IPTW is a statistical approach that compares exposed and unexposed groups in observational studies while adjusting for confounding variables. When we apply the IPTWs to our sample, we create a "pseudosample" in which confounding variables are equally distributed across groups, mimicking the balance that can be achieved in an RCT.

Creating IPTWs involves two steps:

1) The probability (also called the propensity) of being exposed (homeless) is calculated based on measured variables (total chronic health conditions, age, drug use, binge alcohol use, age first homeless, victimization, sex, visiting a regular healthcare provider, getting outpatient mental health treatment, and getting a prescription medication for mental health). This gives the propensity score, which ranges from 0-1.

2) The propensity score is used to calculate an IPT weight for each individual. The IPTWs are calculated as the inverse of the probability of having the actual exposure.

Average Treatment Effects (ATEs)

Using IPTW, we can calculate average treatment effects (ATE). In our observational study, as housing is not a treatment in the traditional sense, the ATE can be thought of as the "average cross-sectional exposure effect" rather than the average treatment effect. In other words, the ATE gives an estimate of how the exposure (homelessness) is associated with the outcome (moderate-severe depressive symptoms).

Augmented Inverse Probability Weighting (AIPTW)

Augmented Inverse Probability Weighting (AIPTW) is a procedure that combines IPTW and ATEs. It is considered "robust" to misspecification and is less subject to bias. This method is straightforward to use and has existed for over 20 years. See references below for more detail.

© 2024 Dobbins SK et al. *JAMA Network Open*

The AIPTW approach includes two steps:

1) Calculate the probability (the propensity) of being exposed (homeless) based on measured variables (total chronic health conditions, age, drug use, binge alcohol use, age first homeless, victimization, sex, visiting a regular healthcare provider, getting outpatient mental health treatment, and getting a prescription medication for mental health). The propensity score is then used to calculate the IPTWs.

2) Estimate two logistic regression models for the outcome of moderate-severe depressive symptoms using IPTWs: One for the exposed condition (homeless), and one for the unexposed condition (housed). The contrast of these two regression models provides the ATE.

Because our exposure variable (residential status) is dichotomous, the treatment effect, or exposure effect, given is the marginal causal odds ratio (MCOR). This can be interpreted as the causal effect of remaining homelessness, compared to regaining housing, on depressive symptoms.

Longitudinal Design

Because the exposure, outcome, and covariates were all measured at each study time point, they are considered time-varying. Using AIPTW allowed us to account for the time-variability of all the variables over the entire study period. AIPTW was used for each time point and the ATEs were averaged across the study period. The standard errors were clustered by individual to account for repeated measurements across time.

eReferences

1. Thoemmes F, Ong AD. A Primer on Inverse Probability of Treatment Weighting and Marginal Structural Models. Emerg Adulthood 2016;4(1):40-59. doi:10.1177/2167696815621645

2. Ho, M., van der Laan, M., Lee, H., Chen, J., Lee, K., Fang, Y., … White, R. (2023). The Current Landscape in Biostatistics of Real-World Data and Evidence: Causal Inference Frameworks for Study Design and Analysis. Statistics in Biopharmaceutical Research, 15(1), 43–56. https://doi-org.ucsf.idm.oclc.org/10.1080/19466315.2021.1883475

3. Austin PC, Stuart EA. Moving towards best practice when using inverse probability of treatment weighting (IPTW) using the propensity score to estimate causal treatment effects in observational studies. Stat Med 2015;34(28):3661-3679. doi:10.1002/sim.6607

4. Glynn AN, Quinn KM. An introduction to the augmented inverse propensity weighted estimator. Polit Anal 2010;18(1):36-56.

5. Kurz CF. Augmented inverse probability weighting and the double robustness property. Med Decis Making 2021:0272989X211027181.

© 2024 Dobbins SK et al. *JAMA Network Open*

eTable 1: Effect of Residential Status on Odds of Moderate-Severe Depressive Symptoms (CES-D ≥ 22) in HOPE-HOME 1 (n=348)			
**ATE [a,b]**	**MCOR (95% Confidence Interval)**	**P-value**	**Weight [b] (Mean, min/max)**
Homeless	1.07 (1.02, 1.11)	0.005	2.11 (1.52/3.00)

[a] Housed is referent group
[b] AIPTWs conditioned on: Chronic health conditions, age first homeless, age at baseline, gender, visit to PCP, outpatient mental health treatment, prescription of mental health medication, any victimization, moderate-high risk substance use, and binge alcohol use
ATE: Average Treatment Effects, AIPTW: Augmented Inverse Probability of Treatment Weights, CES-D: Center for Epidemiologic Studies Depression Scale

© 2024 Dobbins SK et al. *JAMA Network Open*

**eTable 2: Effect of Residential Status on Odds of Moderate-Severe Depressive Symptoms (CES-D≥22) and Outpatient Mental Health Services in HOPE-HOME 1 & 2**			
**ATE [a,b]**	**MCOR (95% Confidence Interval)**	**P-value**	**Weight [b] (Mean, min/max)**
Never Received Outpatient Mental Health Services (n=238)			
Homeless	1.06 (1.01, 1.11)	0.03	2.14 (1.52/3.01)
Received Outpatient Mental Health Services (n=206)			
Homeless	1.11 (1.06, 1.17)	<0.001	2.03 (1.52/2.93)

[a] Housed is referent group
[b] AIPTWs conditioned on: Chronic health conditions, age first homeless, age at baseline, gender, visit to PCP, outpatient mental health treatment, prescription of mental health medication, any victimization, moderate-high risk substance use, and binge alcohol use
ATE: Average Treatment Effects, AIPTW**:** Augmented Inverse Probability of Treatment Weights, CES-D: Center for Epidemiologic Studies Depression Scale

© 2024 Dobbins SK et al. *JAMA Network Open*

**eTable 3: Effect of Residential Status on Odds of Moderate-Severe Depressive Symptoms (CES-D ≥ 22) in HOPE-HOME 1 & 2 (n=444)**			
**ATE [a,b]**	**MCOR (95% Confidence Interval)**	**P-value**	**Weight [b] (Mean, min/max)**
Homeless	1.08 (1.04, 1.11)	<0.001	2.08 (1.51/3.00)
Skilled Nursing Facility	1.05 (0.97, 1.14)	0.25	

[a] Housed is referent group

[b] AIPTWs conditioned on: Chronic health conditions, age first homeless, age at baseline, gender, visit to PCP, outpatient mental health treatment, prescription of mental health medication, any victimization, moderate-high risk substance use, and binge alcohol use

ATE: Average Treatment Effects, AIPTW**:** Augmented Inverse Probability of Treatment Weights, CES-D: Center for Epidemiologic Studies Depression Scale

eTable 4: Effect of Residential Status on Continuous CES-D Depression Score in HOPE-HOME 1 & 2 (N=444)			
**ATE [a, b]**	**Coefficient (95% Confidence Interval)**	**P-value**	**AIPTW Mean (min/max)**
Homeless	3.05 (2.00, 4.13)	<0.001	2.08 (1.51/3.00)

[a] Housed is referent group
[b] AIPTWs conditioned on: Chronic health conditions, age first homeless, age at baseline, gender, visit to PCP, outpatient mental health treatment, prescription of mental health medication, any victimization, moderate-high risk substance use, and binge alcohol use
ATE: Average Treatment Effects AIPTW**:** Augmented Inverse Probability of Treatment Weights, CES-D: Center for Epidemiologic Studies Depression Scal

© 2024 Dobbins SK et al. *JAMA Network Open*

**eTable 5: Effect of Lagged Residential Status on Odds of Moderate-Severe Depressive Symptoms (CES-D ≥ 22) in HOPE-HOME 1 (n=348)**			
**ATE [a,b]**	**MCOR (95% Confidence Interval)**	**P-value**	**Weight [b] (Mean, min/max)**
Homeless	1.05 (1.01, 1.90)	0.019	2.11 (1.52/3.00)

[a] Housed is referent group
[b] AIPTWs conditioned on: Chronic health conditions, age first homeless, age at baseline, gender, visit to PCP, outpatient mental health treatment, prescription of mental health medication, any victimization, moderate-high risk substance use, and binge alcohol use
ATE: Average Treatment Effects, AIPTW**:** Augmented Inverse Probability of Treatment Weights, CES-D: Center for Epidemiologic Studies Depression Scale

© 2024 Dobbins SK et al. *JAMA Network Open*

eTable 6: Effect of Residential Status on Odds of Moderate-Severe Depressive Symptoms (CES-D ≥ 22) with Trends in Depression in HOPE-HOME 1 & 2 (n=444)		
**MCOR (95% Confidence Interval)**	**P-value**	**Weight [b] (Mean, min/max)**
1 year trend 1.06 (1.02, 1.10)	<0.001	2.08 (1.51/3.00)
1.5 years trend 1.07 (1.03, 1.11)	<.01	2.08 (1.51/3.00)
2.5 year trend 1.07 (1.03, 1.11)	<.000	2.08 (1.51/3.00)

[a] Housed is referent group
[b] AIPTWs conditioned on: Chronic health conditions, age first homeless, age at baseline, gender, visit to PCP, outpatient mental health treatment, prescription of mental health medication, any victimization, moderate-high risk substance use, and binge alcohol use
ATE: Average Treatment Effects, AIPTW**:** Augmented Inverse Probability of Treatment Weights, CES-D: Center for Epidemiologic Studies Depression Scale

© 2024 Dobbins SK et al. *JAMA Network Open*

**eTable 7: Effect of Residential Status on Odds of Moderate-Severe Depressive Symptoms (CES-D ≥ 22) with Social Isolation Covariate in HOPE-HOME 1 & 2 (n=444)**			
**ATE [a,b]**	**MCOR (95% Confidence Interval)**	**P-value**	**Weight [b] (Mean, min/max)**
Homeless	1.06 (1.02, 1.10)	<0.01	2.08 (1.51/3.00)

[a] Housed is referent group
[b] AIPTWs conditioned on: Chronic health conditions, age first homeless, age at baseline, gender, visit to PCP, outpatient mental health treatment, prescription of mental health medication, any victimization, moderate-high risk substance use, and binge alcohol use
ATE: Average Treatment Effects, AIPTW**:** Augmented Inverse Probability of Treatment Weights, CES-D: Center for Epidemiologic Studies Depression Scale

© 2024 Dobbins SK et al. *JAMA Network Open*

**eTable 8: Covariate Balance Summary**			
	Standardized Mean Differences		Variance Ratio
	Raw	Weighted	*Weighted*
**Total Chronic Conditions**	-0.1230599	-0.0029396	*1.046743*
**Age at baseline (years)**	0.0359573	0.0001859	*1.031556*
**Moderate-high risk substance use**	0.0039297	1.58E-07	*0.9999979*
**Binge alcohol use**	0.0819953	-0.0036161	*0.9904247*
**Age First Homeless**	0.0108318	0.0037088	*0.8402603*
**Outpatient mental health treatment**	-0.3104121	-0.0020802	*1.000769*
**Saw healthcare provider**	-0.1686649	0.0001189	*1.000203*
**Prescribed mental health medications**	-0.1645643	0.0004139	*1.000609*
**Any Victimization**	0.1513203	0.0068911	*1.004991*
**Male (*vs* Female)**	-0.1244331	0.0017874	*1.001902*
**Overidentification test: chi2 = 14.9755, p=0.1836**			

© 2024 Dobbins SK et al. *JAMA Network Open*

eFigure 1: Number of Follow-up Study Visits Completed

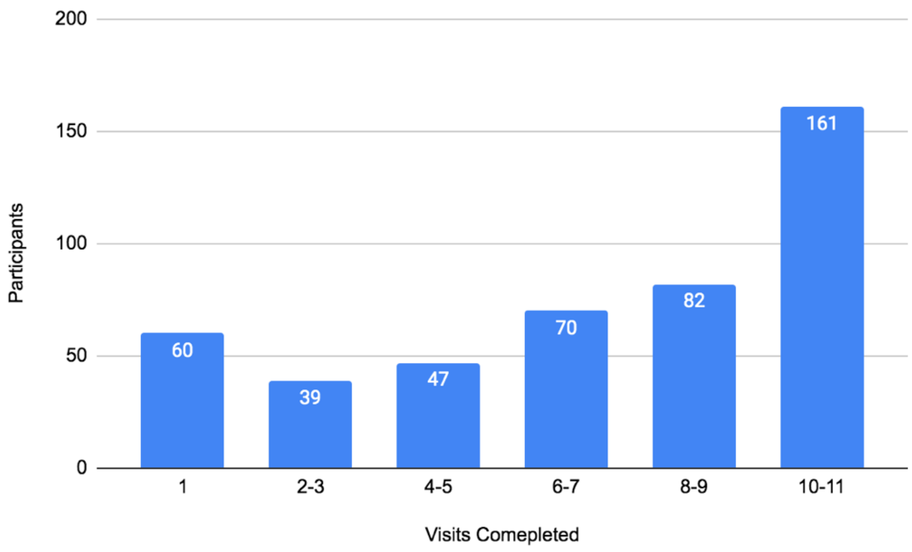

© 2024 Dobbins SK et al. *JAMA Network Open*

eFigure 2: Analysis Sample Diagram

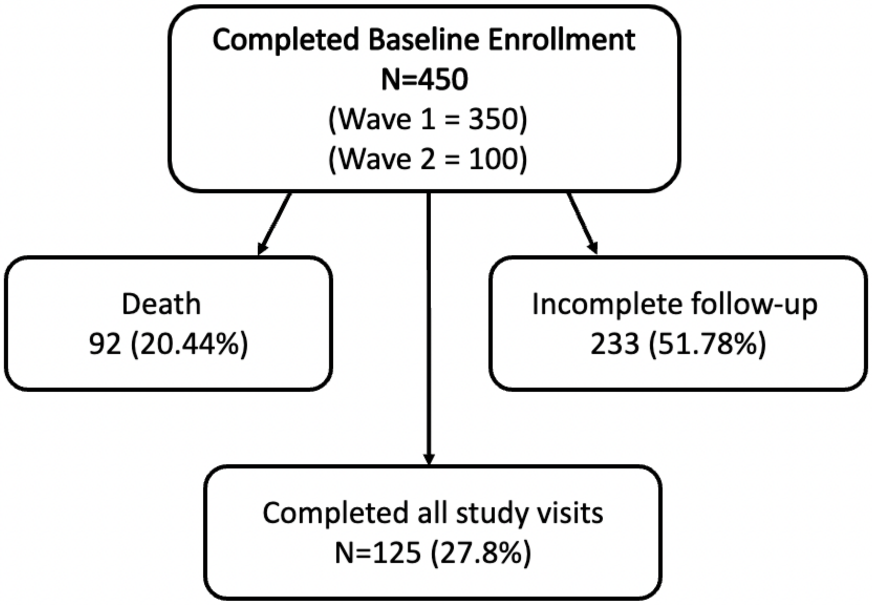

The time period of the analysis includes baseline through 60-month follow-up (5.5 years)

© 2024 Dobbins SK et al. *JAMA Network Open*

eFigure 3: E-value curve

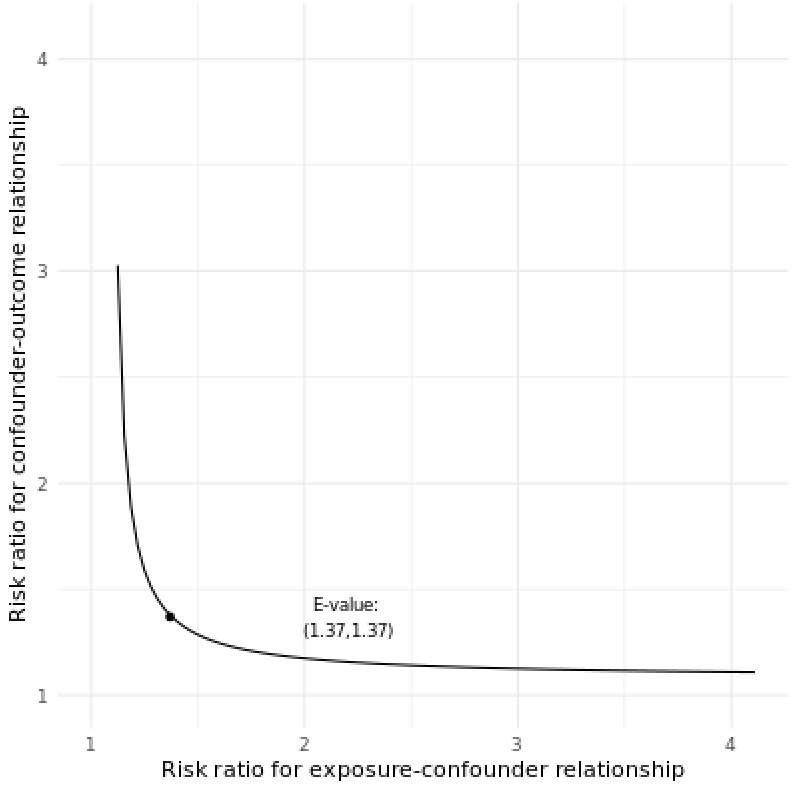

Each point along the curve defines a joint relationship between the two sensitivity parameters that could potentially explain away the estimated effect. A larger E-value implies that considerable unmeasured confounding would be needed to explain away an effect estimate. Although this value is relatively small, we control for several major confounding variables in this study, which increases robustness to unmeasured confounding. In our study, an unmeasured confounder would have to be associated with both homelessness and depressive symptoms by an Odds ratio of 1.37 each.

Reference:

1. Mathur MB, Ding P, Riddell CA, VanderWeele TJ (2018). Website and R package for computing E-values. Epidemiology, 29(5), e45-e47.

eFigure 4: Density Plot of Propensity Scores in AIPTW Treatment Effects Model

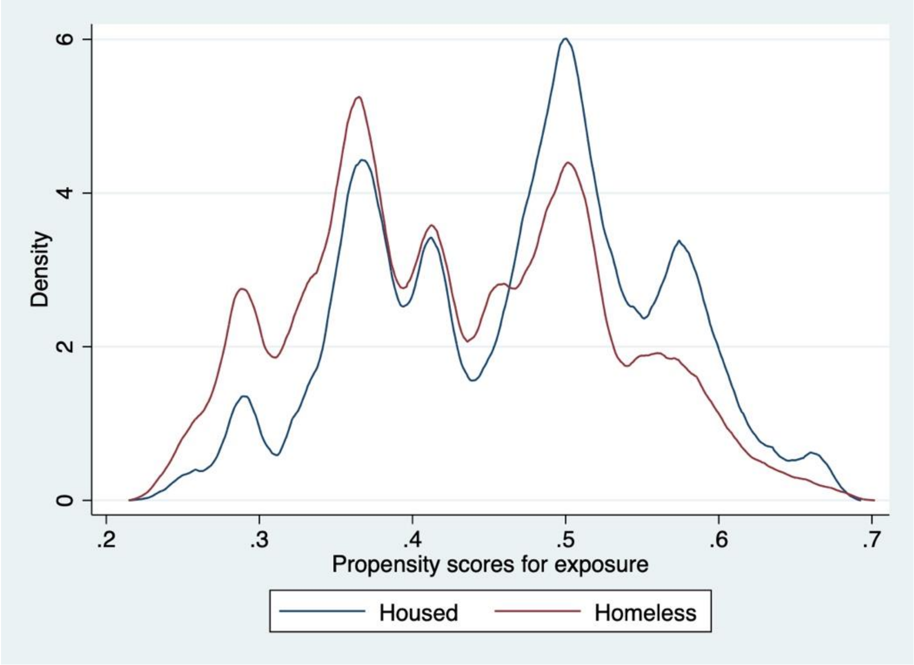

Supplement: Supplement 1. — eMethods eTable 1. Effect of Residential Status on Odds of Moderate-Severe Depressive Symptoms (CES-D ≥22) in HOPE-HOME 1 (n=348) eTable 2. Effect of Residential Status on Odds of Moderate-Severe Depressive Symptoms (CES-D ≥22) and Outpatient Mental Health Services in HOPE-HOME 1 & 2 eTable 3. Effect of Residential Status on Odds of Moderate-Severe Depressive Symptoms (CES-D ≥22) in HOPE-HOME 1 & 2 (n=444) eTable 4. Effect of Residential Status on Continuous CES-D Depression Score in HOPE-HOME 1 & 2 (N=444) eTable 5. Effect of Lagged Residential Status on Odds of Moderate-Severe Depressive Symptoms (CES-D ≥22) in HOPE-HOME 1 (n=348) eTable 6. Effect of Residential Status on Odds of Moderate-Severe Depressive Symptoms (CES-D ≥22) with Trends in Depression in HOPE-HOME 1 & 2 (n=444) eTable 7. Effect of Residential Status on Odds of Moderate-Severe Depressive Symptoms (CES-D ≥22) with Social Isolation Covariate in HOPE-HOME 1 & 2 (n=444) eTable 8. Covariate Balance Summary eFigure 1. Number of Follow-up Study Visits Completed eFigure 2. Analysis Sample Diagram eFigure 3. E-Value Curve eFigure 4. Density Plot of Propensity Scores in AIPTW Treatment Effects Model [file jamanetwopen-e2427956-s001.pdf]
