# Supplementary material for: Continued Homelessness and Depressive Symptoms in Older Adults
Source: JAMA Netw Open. 2024 Aug 15;7(8):e2427956. doi: 10.1001/jamanetworkopen.2024.27956 (PMC11327886; doi:10.1001/jamanetworkopen.2024.27956)
Supplement: Supplement 2. — Data Sharing Statement [file jamanetwopen-e2427956-s002.pdf]

Data Sharing Statement

Dobbins. Continued Homelessness and Depressive Symptoms in Older Adults. *JAMA Netw Open*. Published August 15, 2024. doi:10.1001/jamanetworkopen.2024.27956

Data
Data available: No
